# Men’s Reasons to Abstain from Masturbation May Not Reflect the Conviction of “reboot” Websites

**DOI:** 10.1007/s10508-020-01722-x

**Published:** 2020-04-30

**Authors:** Roland Imhoff, Felix Zimmer

**Affiliations:** Social and Legal Psychology, Department of Psychology, Johannes Gutenberg University Mainz, Binger Str. 14-16, 55122 Mainz, Germany

We recently published a paper titled “Abstinence from Masturbation and Hypersexuality” (Zimmer & Imhoff, 2020) in which we tried to explore correlates of men’s motivation to stay abstinent from masturbation. In motivating the study, we pointed to existing discourses around the topic and cited different protagonists within this debate (e.g., the Web sites “nofap.org” and “rebootnation.org”). Erroneously, one passage might have been unclear in differentiating between the two as it read:

For a period of time known as their “reboot,” the porn-critical subreddit NoFap encourages their followers to abstain from masturbation (“What is NoFap?”, 2018). They assert that “Most guys need to ‘TEMPORARILY’ [sic] eliminate or drastically reduce masturbation and ORGASMS [sic]” (Deem, 2014).

We want to point readers’ attention to the fact that the cited passage does not originate from nofap.org, but from rebootnation.org, another porn-critical forum. In addition, the latter statement was taken out of context as it referred specifically to masturbation reduction as a strategy to overcome pornography-induced erectile dysfunction. We are grateful to several sources for bringing this to our attention, and we want to apologize for the potential confusion of NoFap and Reboot Nation.

It has also come to our attention that some readers may have mistakenly assumed that the 1063 participants in our study were recruited via nofap.org or a similarly named subreddit. This was not the case as we clearly explicated in the paper. It is certainly regrettable that some readers were confused; however, we refute the idea that a scientific text is to be held accountable for how it might be perceived by “casual” readers.

There seems to be a conflict about the interpretation of masturbation and porn abstinence that is highly politicized. Individuals or organizations behind commercial offers (like the community membership offered by NoFap LLC.) have legitimate financial stakes and are thus equally legitimately concerned about their vulnerability to defamation campaigns. As they operate in the public sphere, however, we deem it not only as legitimate but necessary to acknowledge and cite them as one prominent voice in the debate around masturbation abstinence—everything else would be an unjustifiable muting of their stand.

We have no stakes or stands in the dispute (that has culminated in legal action) but were merely interested in the underlying structure of men’s decision to voluntarily refrain from masturbation. Based on more than a thousand responses from a relatively unselected Internet forum, our data suggest that the motivational correlates point stronger in the direction of value conflict and (mis-)perceptions of masturbation as unhealthy than actual problematic sexuality. Clearly, these data rely on self-report rather than objective data and may thus reflect men’s subjective reality more than an “objective” truth. Equally clearly, pointing to Web sites as visible protagonists in the discourse around masturbation abstinence does not imply that our participants were official members of their community.
